# Climate and Demography Dictate the Strength of Predator-Prey Overlap in a Subarctic Marine Ecosystem

**DOI:** 10.1371/journal.pone.0066025

**Published:** 2013-06-18

**Authors:** Mary E. Hunsicker, Lorenzo Ciannelli, Kevin M. Bailey, Stephani Zador, Leif Christian Stige

**Affiliations:** 1 College of Earth, Ocean and Atmospheric Sciences, Oregon State University, Corvallis, Oregon, United States of America; 2 Alaska Fisheries Science Center, National Marine Fisheries Service, National Oceanic and Atmospheric Administration, Seattle, Washington, United States of America; 3 Centre for Ecological and Evolutionary Synthesis, Department of Biology, University of Oslo, Oslo, Norway; The Australian National University, Australia

## Abstract

There is growing evidence that climate and anthropogenic influences on marine ecosystems are largely manifested by changes in species spatial dynamics. However, less is known about how shifts in species distributions might alter predator-prey overlap and the dynamics of prey populations. We developed a general approach to quantify species spatial overlap and identify the biotic and abiotic variables that dictate the strength of overlap. We used this method to test the hypothesis that population abundance and temperature have a synergistic effect on the spatial overlap of arrowtooth flounder (predator) and juvenile Alaska walleye pollock (prey, age-1) in the eastern Bering Sea. Our analyses indicate that (1) flounder abundance and temperature are key variables dictating the strength of flounder and pollock overlap, (2) changes in the magnitude of overlap may be largely driven by density-dependent habitat selection of flounder, and (3) species overlap is negatively correlated to juvenile pollock recruitment when flounder biomass is high. Overall, our findings suggest that continued increases in flounder abundance coupled with the predicted long-term warming of ocean temperatures could have important implications for the predator-prey dynamics of arrowtooth flounder and juvenile pollock. The approach used in this study is valuable for identifying potential consequences of climate variability and exploitation on species spatial dynamics and interactions in many marine ecosystems.

## Introduction

Climate- and human-induced changes in marine ecosystems have detectable impacts on species spatial dynamics [1]–[3]. The potential effects on marine food webs are of increasing interest. For example, different responses of predators and prey to changing climate conditions decouple species interactions [4], and diminished thermal boundaries cause the establishment of new and amplified trophic linkages [5]. In addition to climate-driven variability, density-dependent (i.e. demographic) variables can affect species spatial dynamics [6]–[8] and thereby impact predator-prey interactions. While much progress has been made towards understanding the causes of changes in species distributions, little is known about how these shifts influence the strength of predator-prey overlap and prey population dynamics.

**Figure 1:**
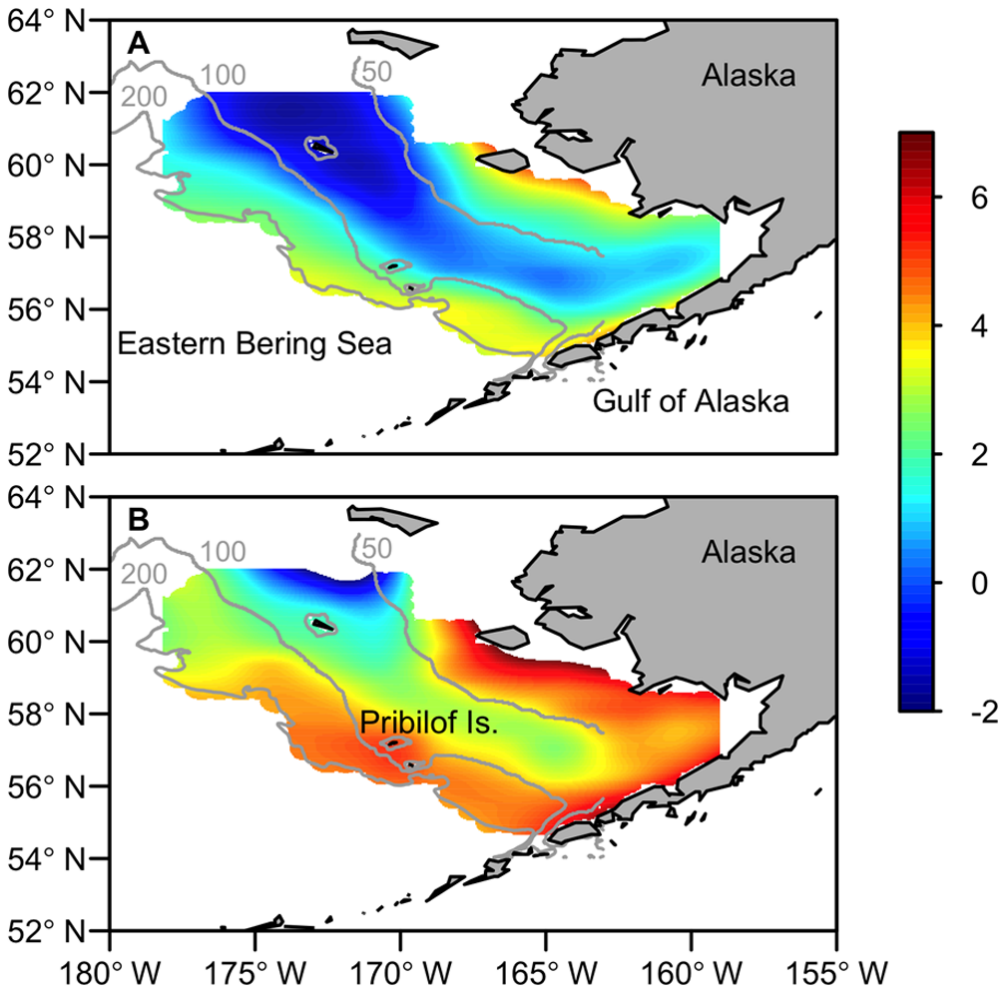
Study region and cold pool. Summer survey bottom temperatures (°C) in the eastern Bering Sea during a cold year (A; 2007) and warm year (B; 2003). The 50 m, 100 m and 200 m depth contours are shown.

The potential for changes in species distributions and interactions is pronounced in subarctic ecosystems. These systems support some of the largest fisheries in the northern hemisphere. Also, climate-induced habitat variability is especially strong in subarctic systems, which are at the boundaries of different biogeographic zones and are heavily influenced by seasonal sea-ice. For example, in the eastern Bering Sea the spatial distributions of several species are strongly influenced by water temperatures and the location of a cold pool of subsurface water (<2°C) that forms across the middle region of the continental shelf due to the formation and melting of winter sea-ice (Fig. 1; [9]–[11]). In cold years, species that are intolerant of low temperatures are often restricted to warmer waters along the outer continental shelf and slope regions. This reduction in suitable habitat leads to higher spatial concentrations of cold-intolerant predators and prey. In warm years species distributions are less constrained by thermal conditions. Species or particular life stages that utilize the cold pool as a refuge are more vulnerable to predators and competitors during warm periods (e.g. [5]).

**Figure 2:**
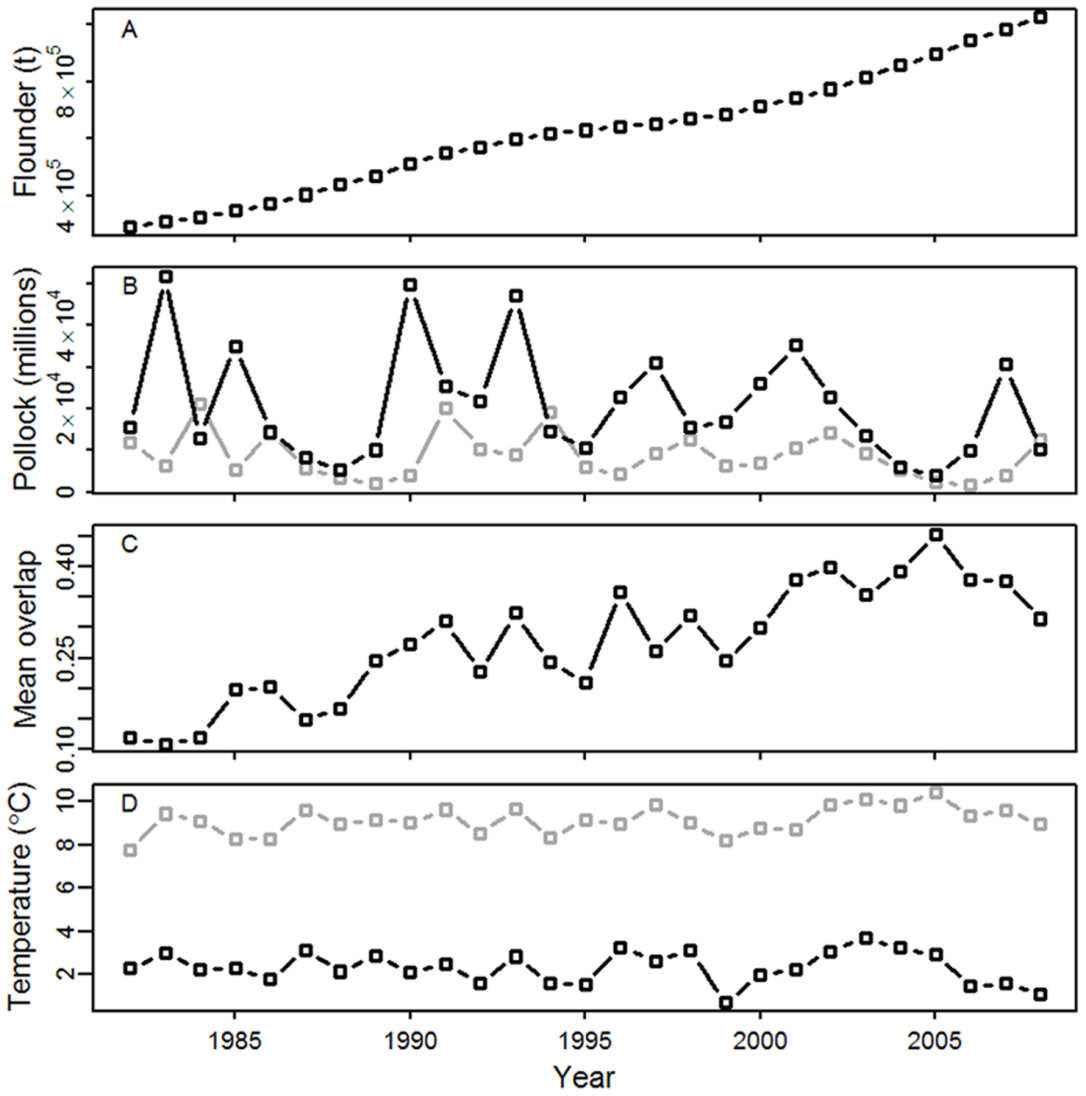
Time series data. Annual estimates of flounder biomass (A; in tons), age-1 pollock abundance (B; black line, in millions), age-2 pollock abundance (B; gray line, in millions), mean probability of flounder and age-1 pollock overlap (C; based on model estimates), mean July to September sea surface temperatures (D; gray line), and mean summer trawl survey bottom temperature (D; black line).

In recent years, the eastern Bering Sea has also been the stage for profound changes in species abundance and distribution [8], [11]–[12]. For example, the abundance of arrowtooth flounder (*Atheresthes stomias*, hereafter referred to as flounder) has increased eight-fold over the past three decades (Fig. 2a, [13]); furthermore, the nonadditive effects of high biomass and warm water temperatures have prompted their expansion into new habitat [8]. We may expect that the rising trend in flounder biomass coupled with the predicted long-term warming of water temperatures in the eastern Bering Sea [14], [15] will have an important influence on the strength of spatial overlap between flounder and its prey. Of particular interest is the potential impact of increased flounder predation on the population dynamics of Alaska walleye pollock (*Theragra chalcogramma*, hereafter referred to as pollock). Pollock dominates the groundfish biomass in the Bering Sea and is both ecologically and economically valuable in this system [16]. Similar to flounder, the spatial dynamics of pollock are influenced by water temperature [17], [18]. Thus, changing ocean conditions could affect flounder and pollock spatial overlap through temperature-induced shifts in prey distributions as well.

The documented effects of environmental and demographic variables on species distributions raise an important question: how are trophic linkages altered by changes in species spatial dynamics? The Bering Sea is an ideal system to examine the ecological consequences of changes in species distribution. Like other heavily harvested systems, the Bering Sea is the focus of intense assessment surveys aimed at estimating species abundance, distribution and trophic interactions. The survey data provide an opportunity to improve our understanding of species spatial dynamics and ecological interactions in sub-arctic ecosystems. Using these data, we developed a spatially explicit approach to identify the potential mechanisms that dictate the location and magnitude of predator and prey overlap. Because the intensity of species interactions depend on the strength of their overlap, it is necessary to first quantify how environmental conditions and demography influence the spatial overlap between trophically connected species. Previously described indices of spatial overlap often express central tendency (e.g. Schoener [19], Williamson [20]) and are not spatially explicit. However, our methodology provides the ability to characterize the dynamics of species interactions and quantify the impact of predators on prey across space under different scenarios. We applied this approach to flounder (predator) and age-1 pollock (prey) to test the hypothesis that population size and ocean temperatures have a synergistic effect on the spatial overlap of these species. In addition, we examined whether the magnitude of species overlap could potentially influence the survival of age-1 pollock. Flounder is a major predator of pollock, especially the age-1 individuals (hereafter referred to as juvenile pollock; [12]). Therefore, we focus on this life stage in our study.

## Methods

For our analyses, we used biological data collected from standard annual bottom trawl surveys conducted by the National Oceanic and Atmospheric Administration (NOAA) Alaska Fisheries Science Center (AFSC; Seattle, Washington) groundfish assessment program. Species weight, number, and length and environmental data are routinely recorded during survey trawl sampling. The survey stations are located within a 37×37 km grid pattern (fixed stations) where bottom depths range from 50 m to 200 m. Sampling starts between late May and early June and ends in late July or early August. Only successful trawls meeting AFSC standardized performance criteria were selected for this study. Also, there is some variability in the number of survey stations sampled each year and we restricted our analysis to stations that were sampled during at least 24 years between 1982 and 2008. The average number of stations used in this study was 281 (±15 SD). Details on trawl survey gear/methods and sampling design can be found in [21] and [22], respectively.

We used a two-step approach to quantify the effects of environmental and demographic variables on the location and magnitude of overlap between adult flounder (>20 cm) and juvenile pollock (≤20 cm, which is equivalent to age-1 in this study because age-0 are rarely captured in the trawl survey, [16]). First, we modeled the presence/absence of flounder and pollock in relation to biotic and abiotic indices. Species distributions were modeled separately as binomial probability functions with the logit link function in variable coefficient generalized additive models (GAM). A key assumption of the variable coefficient GAM is that the relationship between species occurrence and model covariates is spatially variable and locally linear (at the scale of the predictor variables). Second, we calculated the joint probability of species spatial occurrence by multiplying model predictions of flounder and pollock spatial occurrence. The joint probabilities were used as a metric to identify how the location and magnitude of species spatial overlap might change in relation to shifts in environmental and demographic conditions. We note that we modeled species occurrence rather than abundance because there is low catchability of juvenile pollock in the trawl surveys [16]; therefore the presence/absence information is less biased than estimates of local abundance.

To model flounder and pollock distributions we implemented two types of model formulations. For flounder, we used a non-stationary GAM, where the additive and nonadditive effects of a covariate(s) are assumed to change abruptly over time at a threshold value [8], [23]–[24]. Flounder biomass has increased consistently in the eastern Bering Sea over the past 30 years (Fig. 2a, [13]) and Ciannelli and others [8] found that the trend in population biomass had a threshold effect on the species spatial dynamics in this system. The authors showed that when flounder biomass was below the threshold estimated by the data, the species occupancy was mostly affected by water temperature. When flounder biomass was above the threshold, the species occupancy was regulated by both water temperature and their population biomass. Their study provides further evidence of regime shifts in ecological systems [25], [26].

We used a model formulation similar to Ciannelli and others [8] to capture the threshold effect of flounder biomass on the occurrence of flounder. The full model formulation for flounder is as follows:
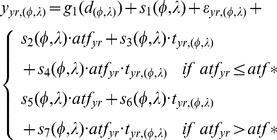
(1)where *y* is the logit ( = ln(P/(1−P)) of the probability (P) of presence of adult flounder in year *yr* at given location, *φ* is latitude, *λ* is longitude, *d* is bottom depth in meters, *t* is bottom (gear) temperatures, *atf* is the estimated annual biomass of flounder (age 1+), atf* is the threshold value of flounder biomass estimated from the data, ε is an error term, and *g* and *s* indicate univariate and two-dimensional smoothing functions (thin plate regressions splines, [27]). The threshold value of flounder biomass (atf*) was estimated by minimizing the Akaike Information Criterion (AIC, [28]) among full models spanning the range of potential threshold values (see [8] for details). We restricted the range of values to the upper 0.8 and lower 0.2 quantiles to guarantee that at least 20% of the available sample size was included below or above candidate threshold values [23].

Exploratory analysis indicated that the spatial dynamics of juvenile pollock did not change abruptly at a single threshold level of pollock biomass or temperature. Thus, we modeled the distribution of juvenile pollock using a stationary formulation, i.e. the additive and non-additive effects of a covariate(s) on the occurrence of pollock were assumed to be similar over time. The full model formulation for juvenile pollock is as follows:
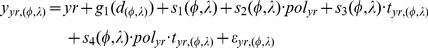
(2)where *y* is the logit of the probability of presence of juvenile pollock (age-1, ≤20 cm) in year *yr* (as factor) and *pol* is the estimated annual number of juvenile pollock. Stock assessment reports provided estimates of annual flounder population biomass [13] and pollock abundance [16]. Bottom temperature and depth were recorded at each trawl survey location with high precision temperature and depth profilers (Sea-Bird SBE-39 data recorder; Sea-Bird Electronics, Bellevue, Washington, USA). Prior to analysis we standardized estimates of flounder biomass, juvenile pollock abundance, and bottom temperatures (by subtracting the mean and dividing by standard error). We note that we did not include spatial terms for flounder and pollock in the other species candidate models because the inclusion of the predator and prey terms has potentially confounding effects on the estimates of species overlap and the recruitment analysis. However, we did quantify the effects of the flounder and pollock on the species distribution to check if we missed important parts of the dynamics (see Models 1A and 5A in Table S1).

We compared the full model formulations for both species (equations 1 and 2) to reduced versions to identify the level of model complexity that best explained the variance in the response (see Tables 1 and S1). In the presence of positive residual autocorrelation, AIC-selection would likely lead to an overly complex model. Thus, we also used genuine Cross Validation (gCV) to account for spatial autocorrelation and determine which level of model complexity was optimal for prediction. To do so, we randomly split our dataset into training and validation data (70% and 30% of the dataset, respectively). We then fit models to the training data, generated predictions for the validation data, and calculated the prediction error. The mean squared prediction error was used as the gCV criterion. The candidate model with the lowest AIC and gCV value was deemed the best model for predicting species occurrence. In the case that multiple models had similarly low AIC and gCV measures, the most parsimonious model (i.e. least number of parameters) was used for making predictions. The mgcv library (version 1.17–22, [27]) in R (version 2.15.2, [29]) was used for model construction and comparisons.

**Table 1:** GAM structures and selection criteria. See methods for description of model terms and notations.

ID	Models	Dev expl	UBRE	AIC	gCV
	*Juvenile pollock*				
1	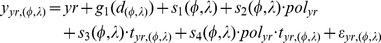	20.6	−0.030	7365	0.1595
2	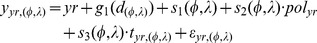	20.5	−0.029	7362	0.1597
3	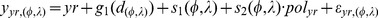	19.7	−0.250	7392	0.1603
**4**	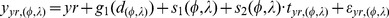	**20.4**	−**0.029**	**7362**	**0.1595**
	*Arrowtooth flounder*				
**5**	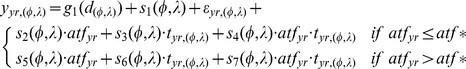	**73.6**	−**0.604**	**3005**	**0.0614**
6	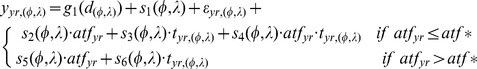	72.1	−0.589	3117	0.0641
7	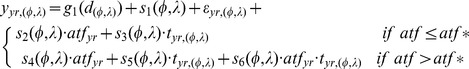	72.8	−0.595	3070	0.0633
8	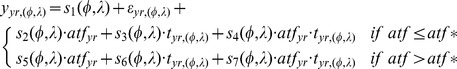	72.5	−0.597	3057	0.0618

Models with IDs and selection criteria highlighted in bold best predicted the occurrence of adult flounder and juvenile pollock in the eastern Bering Sea. Dev expl, % deviance explained; UBRE, Un-Biased Risk Estimator criterion; AIC, Akaike Information Criterion; gCV, genuine cross validation.

From the best fit model for each species, we predicted probabilities (P) of flounder and pollock occurrence at all survey locations (*φ,λ*) in all years (*yr*). Species overlap was quantified by multiplying the predicted probabilities of species occurrence, i.e. P_overlap,*(φ,λ),yr*_ = P_atf,*(φ,λ),yr*_ * P_pol,*(φ,λ),yr*_. We then identified how the magnitude and location of the overlap metric varied in relation to environmental and demographic conditions. We quantified the change in species overlap under warm and cold ocean conditions during periods of low and high flounder biomass. To do this, we selected data from years 1985 and 2005, which represent warm years and regimes of low and high flounder biomass, respectively. Next, we calculated the predicted values of species occurrence (using best fit models) and species overlap during each regime, with and without a standardized unit decrease in water temperature at all survey locations. The differences between the predicted values under the different scenarios indicate how species occurrence and overlap might change under alternative environmental and demographic conditions.

Lastly, we examined whether the strength of species overlap might influence the recruitment of juvenile pollock to the age-2 year class. We calculated 15-year moving window correlations between the residuals of a stock-recruitment relationship for age-2 pollock and biotic and abiotic indices. The indices included adult flounder stock size, sea surface and bottom temperatures, and mean model estimates of flounder and juvenile pollock overlap (Fig. 2). The moving window method allowed us to identify any non-stationary correlations. Also, by using stock-recruitment residuals in this analysis we were able to remove the effect of spawning stock biomass on recruitment. Numbers of age-2 pollock [16] were used as an index of age-1 recruitment to the following year class, and estimates of pollock spawning stock biomass [16] were lagged by 2 years to match the age-2 year class. The residuals were estimated using a Ricker stock-recruitment model [30]. The Ricker model is routinely used to assess pollock stock status [16] and to examine the relationship between pollock recruitment and environmental indices (e.g. [31]). To calculate correlations, the estimates of species overlap and flounder biomass were lagged by 1 year because flounder preys heavily on age-1 pollock in summer [12]. Average July to September sea surface temperatures (SST; [32]) and bottom temperatures were also lagged by 1 year to capture the influence of summer temperatures on recruitment [31]. SST was included in this analysis because it has an important influence on pollock recruitment to the age-1 year class. It is likely that the SST time series captures an array of factors that influence recruitment. The stock recruitment residuals and abundance estimates were transformed with natural logs prior to analysis.

## Results

The occurrence of flounder in the eastern Bering Sea was best predicted by the full version of the nonstationary GAM (i.e. lowest AIC and gCV values; Model 5, Tables 1). The nonstationary model outperformed models without a threshold formulation (Table S1). Model selection of full models spanning the range of potential threshold values indicated that flounder biomass had a single threshold effect on this species occurrence when the standardized population biomass was approximately 1.62, which is equivalent to 630,000 t (Fig. S1). Because flounder biomass has been continually increasing over the past few decades, the threshold biomass estimated from the data essentially split the time series into two regimes: a low flounder biomass regime spanning years 1982–1995 and a high flounder biomass regime during years 1996–2008. In both regimes, the interaction of flounder biomass and bottom temperature was an important predictor of flounder occurrence.

Based on the AIC and gCV measures, there was not a clear best fit model for the occurrence of juvenile pollock. Instead, the full and reduced versions of the stationary GAM (Models 1, 2, 4; Table 1) fit the pollock data equally well. For prediction purposes, we selected the most parsimonious model as the best fit model, which includes year, location, depth, and bottom temperature (Model 4; Tables 1 and S2). In general, the model fit for flounder was much stronger than the model fit for juvenile pollock. However, based on visual inspection, the predicted probabilities of species occurrences, as well as spatial overlap, are in agreement with survey catch data for these species. The occurrence of flounder was highest along the outer shelf and slope and southeast shelf regions, whereas juvenile pollock were more broadly distributed with high occurrences along the outer shelf and in the middle and inner shelf regions (Fig. S2). We do not report confidence intervals around the predictions, as the nominal confidence intervals are likely too narrow because of positive spatial autocorrelation. The quantitative values of species occurrence and overlap should be interpreted with some caution, but the cross validation suggests that the results are qualitatively correct.

**Figure 3:**
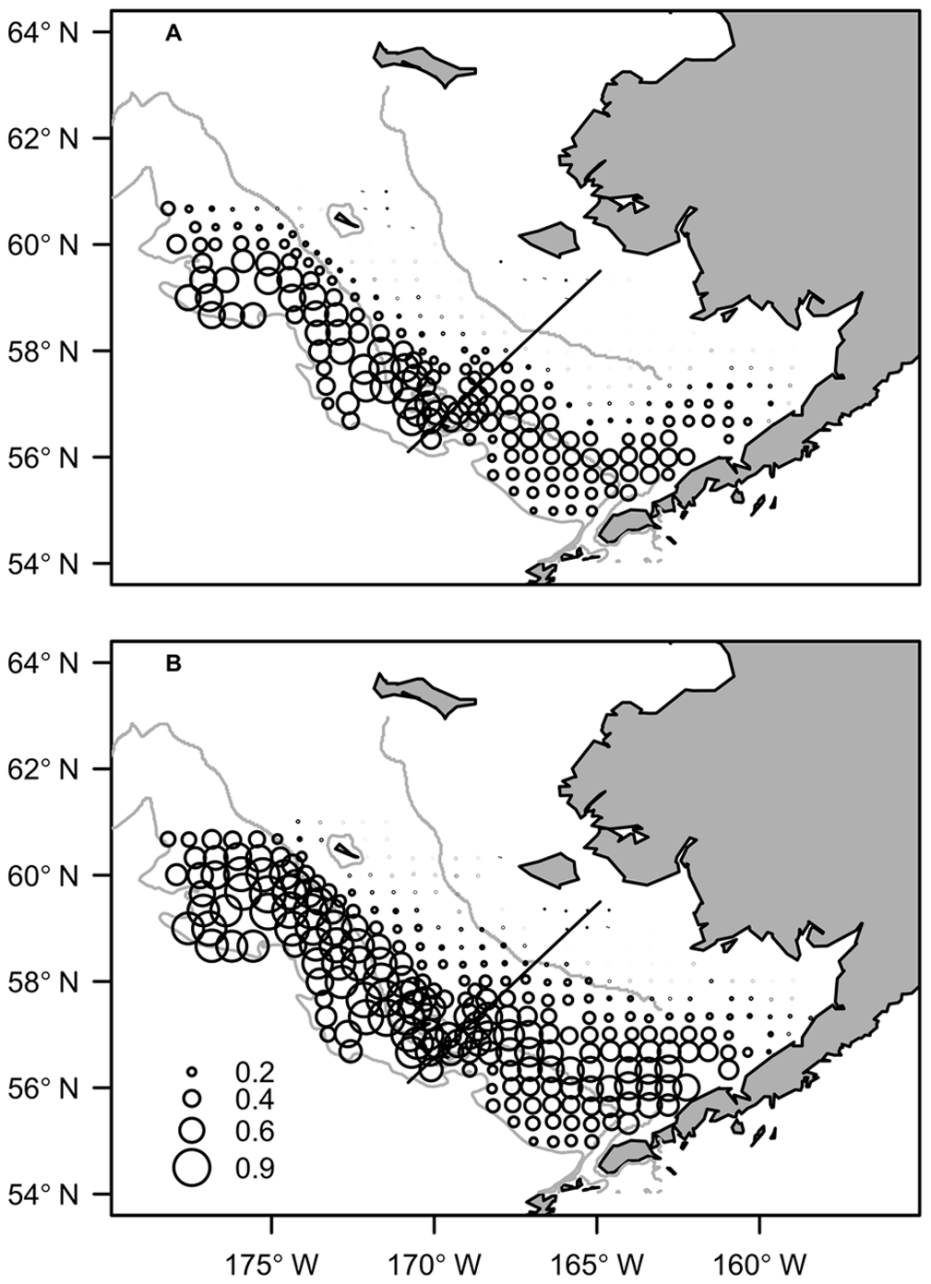
Predicted probability of species overlap. The mean predicted probability of overlap between adult flounder and age-1 pollock during years of low flounder biomass (A; before 1995) and high flounder biomass (B; 1995 and after). Circle size reflects probability of species overlap. Black line represents the north-south line, which delineates the northwest and southeast regions of survey strata [33]. The 50 m, 100 m and 200 m depth contours are shown.

The model predictions indicate that the magnitude of flounder and pollock overlap has increased with the rise in flounder abundance over time. In addition, the predicted probabilities of overlap were higher in the northern region of the continental shelf compared to the southern region (Fig. 3). During the low flounder biomass regime (i.e. 1995 and before), the mean probability of species overlap was highest around and north of the north-south line that is used by NOAA to delineate northwest and southeast portions of the survey strata (Fig. 3a, [33]). In the high flounder biomass regime (i.e. after 1995), there was an expansion in both the magnitude and geographic extent of flounder and juvenile pollock overlap (Fig. 3b). In particular, there was a notable increase in the probability of species overlap in the northwest and southeastern regions of the shelf.

**Figure 4:**
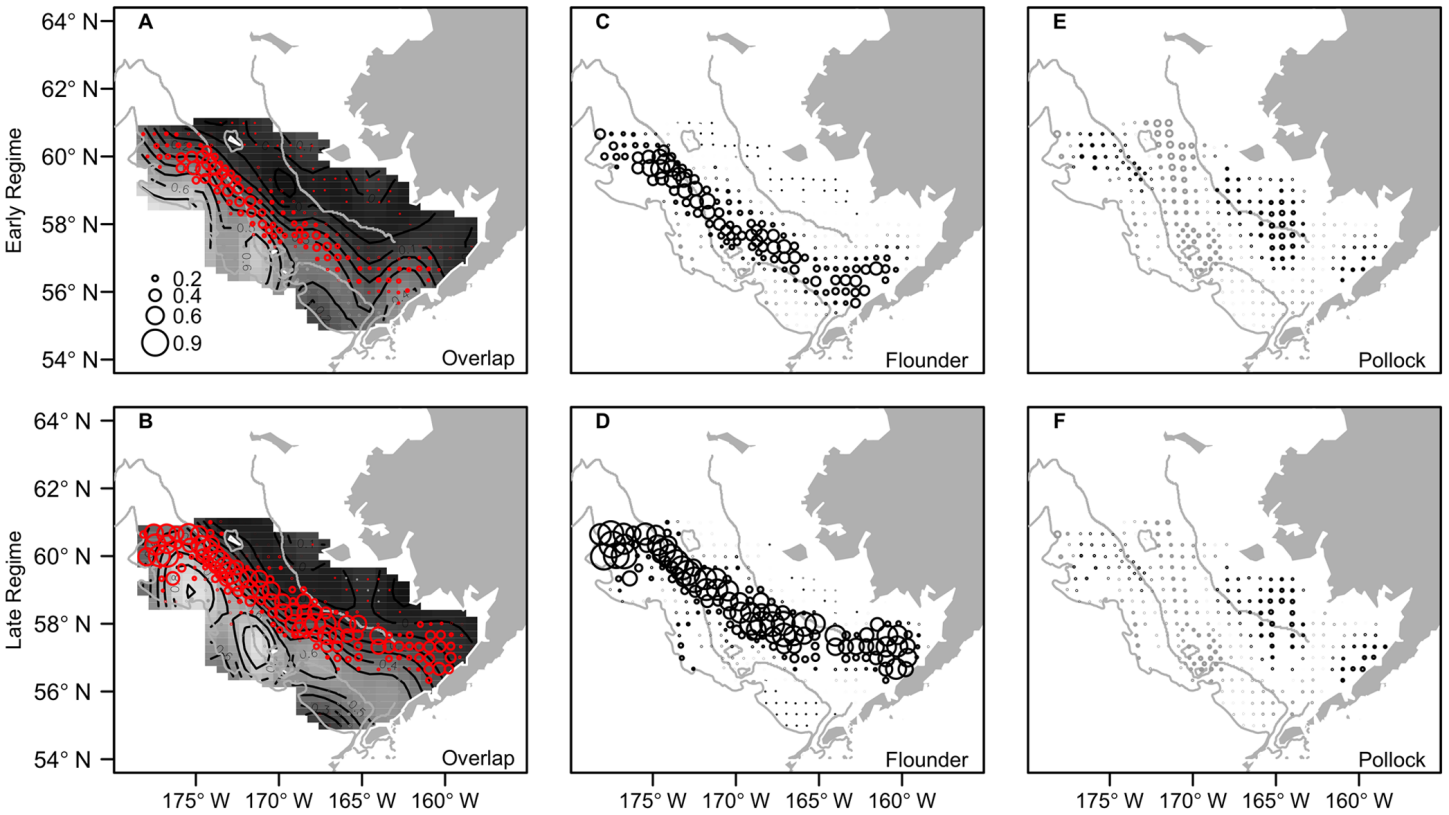
Predicted changes in species overlap with increasing temperatures. Predicted increases in predator and prey occurrences (black circles) and probability of species overlap (red circles) with a standardized unit increase in spatially explicit bottom temperatures in the early regime (based on year 1987; A, overlap; C, flounder; E, juvenile pollock) and late regime (based on year 2005; B, overlap; D, flounder; F, juvenile pollock). Gray circles indicate locations of predicted decreases in species occurrences and overlap with increased temperatures. Circle size reflects magnitude of change. Missing circles at survey locations indicate no change with increasing temperatures. The shaded regions and black isolines (A, B) reflect the probability of overlap averaged across all years in the two regimes under normal conditions, i.e. without an increase in water temperature. Light and dark shading show areas of high and low overlap, respectively. The 50 m, 100 m and 200 m depth contours are shown.

The effect of temperature on the magnitude of species overlap was amplified by high flounder biomass. This is demonstrated by the predicted change in species overlap between cold and warm ocean conditions in low and high flounder biomass regimes (Fig. 4). For example, when flounder biomass was low, the greatest change in overlap with an increase in temperatures occurred mostly along the 100 m isobath in the north shelf region (Fig. 4a). However, when the flounder stock size and temperatures were high, there were large increases in overlap in the northwest shelf and throughout most of the middle and southeast shelf regions (Fig. 4b). In both periods, the predictions of species overlap appeared to be largely driven by the movement of flounder. For example, the greatest increases in overlap occurred in the same regions where flounder occupancy was predicted to increase the most in response to higher temperatures (Fig. 4c,d). We also found that species overlap and flounder occupancy did not change or decreased only slightly in outer shelf regions with increased water temperatures and biomass. This indicates that as flounder move inshore their occurrence and overlap may still remain high along the outer shelf region, or similar to that observed under the original temperature conditions (see shaded regions in Fig. 4a,b). Pollock movement appeared to contribute to increases in species overlap in the northern and southeast shelf in warm years (Fig. 4e,f). However, predicted changes in the occurrence of pollock in response to increased temperature were relatively small compared to those of flounder. The predicted spatial patterns shown for years 1987 and 2005 in figure 4 are representative of other years in the time series. We made corresponding plots to figure 4 for several years in both regimes (not shown here) and the spatial effects of temperature on species distributions and overlap were the same or similar.

**Figure 5:**
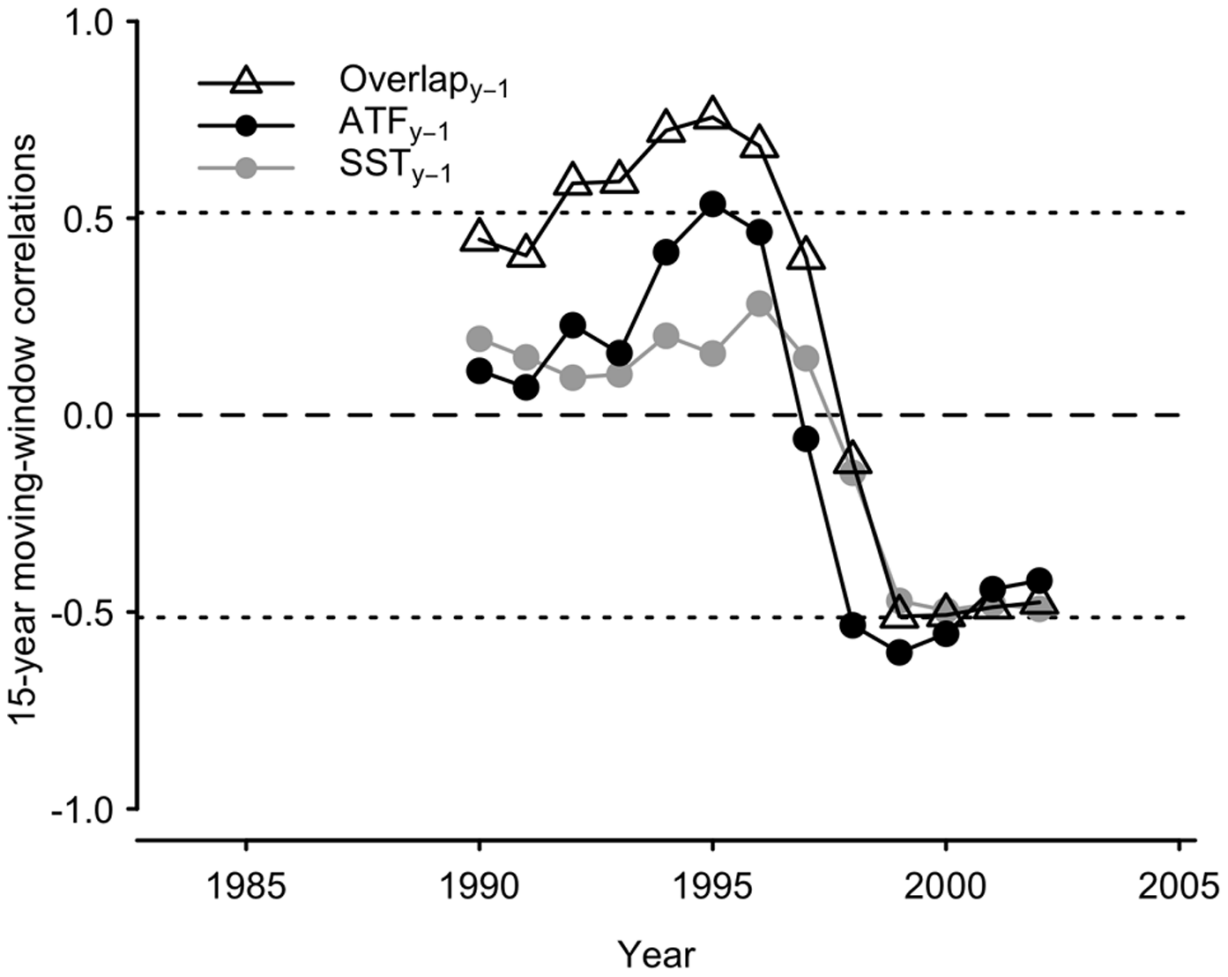
Temporal change in correlations between residuals of pollock stock-recruitment relationship and biotic and abiotic indices. Correlation coefficients were calculated for 15-year moving windows centered at the x-axis values. The symbols indicate the correlations with annual mean flounder and pollock overlap (Overlap_yr−1_), ln-transformed flounder stock size (Flounder_y-1_), and average July-September sea surface temperatures (SST_yr−1,_ °C). Stippled lines: statistical significance of correlations = 0.05 (ignoring autocorrelation).

The moving window correlations of juvenile pollock recruitment indicate the presence of non-stationary relationships. Flounder biomass was weakly correlated with pollock recruitment in the early part of the time series, but exhibited significant, negative correlations after 1997 (Fig. 5). Species overlap and sea surface temperatures (July to September) showed a similar trend. There were strong, negative correlations between these indices and juvenile recruitment after 1998 (Fig. 5). The correlations between bottom temperature and recruitment also shifted from positive to negative over time, but overall they were relatively weak (Fig. S3). Interestingly, species overlap showed a positive and significant correlation with juvenile recruitment in the early and mid-1990s. It is possible that during that period year-to-year variation in overlap was largely influenced by variation in age-1 pollock abundance and that recruitment was to a large extent dictated by survival up to the age-1 life stage. The overlap index may act as a proxy for pre-age-1 survival early in the times series and for predation pressure in the latter period.

## Discussion

Better knowledge of the mechanisms that influence the strength of species overlap improves our ability to anticipate shifts in trophic linkages and forecast ecosystem-level effects of changing environmental conditions. We used a spatially explicit modeling approach to test the effects of population size and water temperature on the location and magnitude of overlap between flounder and juvenile pollock. Our analyses showed that flounder biomass and temperature are important drivers of species overlap and that predicted changes in overlap strength were largely a consequence of flounder movement. Further, we found a negative relationship between juvenile pollock survival and the strength of predator-prey overlap at high levels of flounder biomass. Our findings contribute to the growing evidence that continued increases in flounder abundance combined with warming ocean temperatures could translate into higher predation mortality on juvenile pollock.

**Figure 6:**
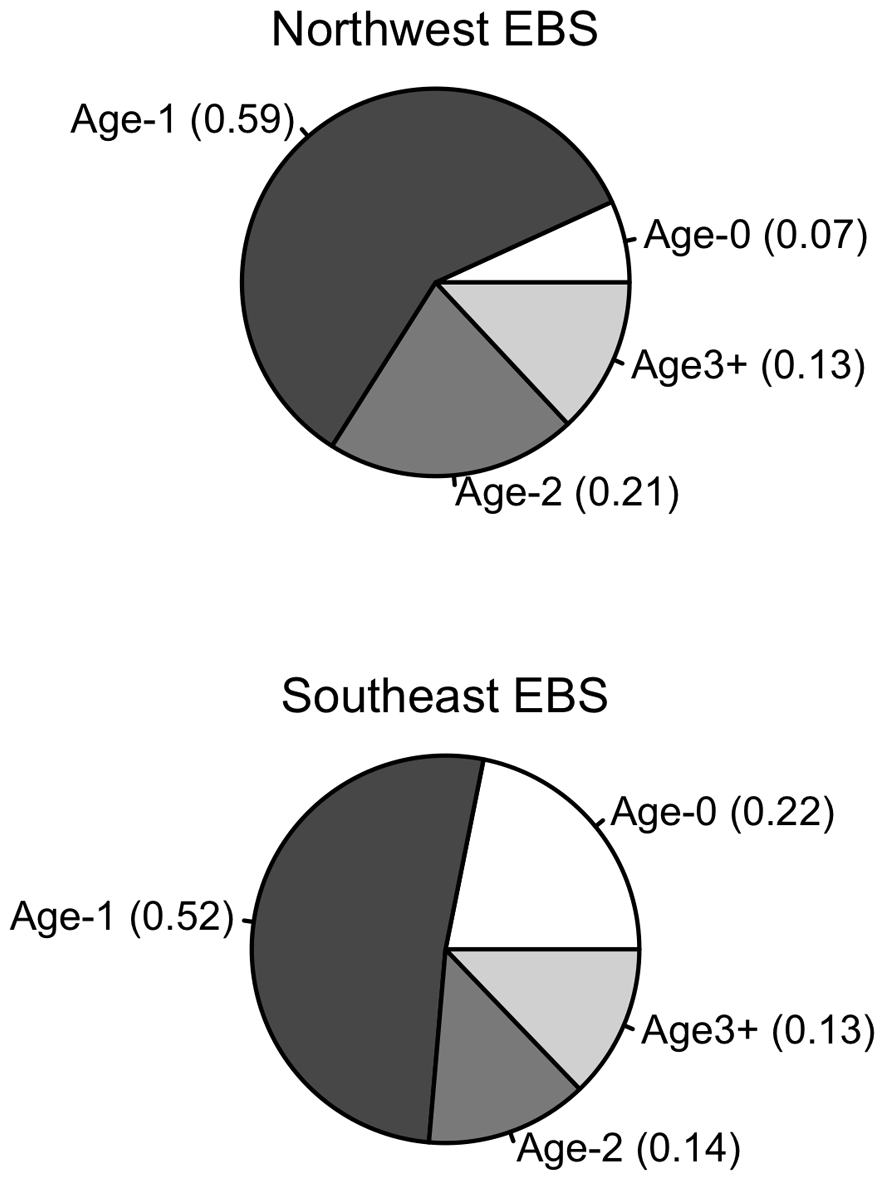
Flounder diet composition. Pollock age-class composition in flounder diets by number for northwest and southeast shelf regions of the eastern Bering Sea (EBS; see Fig. 3 for delineation). The estimated contributions of individual age classes by mass are less certain and more biased than the numerical estimates for each class and therefore are not presented here. The total contributions of pollock to flounder diets in the northwest region are 8% by number, 77% by mass, 28% by occurrence, and in the southeast region, 4% by number, 58% by mass, and 11% by occurrence. Modified from the work of Zador and others [12]. See [12] for details on sampling and analysis.

Characterizing species interactions across space and time is important to properly quantify the impact of predators on prey populations. Our results illustrate that the magnitude of species overlap is spatially and temporally variable. The intensity of species interactions is likely to reflect this variability. Higher overlap between flounder and pollock in the northwest regions of the eastern Bering Sea shelf could have important consequences for the fully exploited pollock population. For example, the pollock fishery has shifted toward the northwest region of the eastern Bering Sea in recent years [16]. In addition, our estimates of species overlap combined with flounder prey composition (Fig. 6) suggest that flounder could exert increased predation mortality on juvenile pollock in this region in coming years. Zador and others [12] made a similar conclusion based on observations of larger flounder body sizes and higher rates of non-empty stomachs in the northern shelf region. While juvenile pollock is an important diet item of flounder throughout the eastern Bering Sea (Fig. 6), the authors propose that current predation on pollock may be highest in the northwest region [12]. Together, these findings warrant the need for more work to accurately estimate juvenile pollock predation mortality to ensure that pollock can support the demands of their predators and the fishery without affecting the population’s resilience. For example, a valuable extension of this study could be to couple our estimated probabilities of overlap with expected local densities of predator and prey and diet composition to quantify the interaction strengths of flounder and pollock over space. It may be prudent to account for the spatial heterogeneity in predation mortality when estimating how much catch the population can sustain. Such spatial management practices may be particularly relevant for stocks that have a complex demographic structure. Pollock in the Bering Sea is likely made of several demographic substocks (although not necessarily genetically distinct stocks, [34]).

The predatory impact of flounder on juvenile pollock could become amplified if increases in flounder abundance and ocean temperatures persist. In the past, cannibalism was a major component of pollock inter-annual recruitment variability [35] whereas flounder played a less important role. However, our model predictions indicate that species overlap might increase throughout the northwest and middle shelf regions with increases in water temperatures. Further, we found evidence of a negative correlation between the survival of juvenile pollock and the magnitude of predator-prey overlap when flounder biomass is high. These findings are consistent with the recent study by Mueter and others [31] that showed that there is a negative relationship between flounder biomass and survival of pollock from spawning to age-1. In addition, those authors forecasted pollock recruitment under different temperature and predator biomass scenarios and found that further increases in flounder biomass could have a strong impact on future pollock recruitment. An important assumption in their calculation of flounder predation is that the proportion of pollock in flounder diets remains stationary over space and time. This assumption is likely to be violated given the heterogeneous patterns of species overlap identified in the present study. Developing projection models that account for the influence of temperature and demography on the magnitude of flounder and pollock overlap could reveal a more realistic picture of how changes in ocean conditions and flounder stock size might affect pollock recruitment.

There are multiple factors that can affect the availability of juvenile pollock to predatory flounder. For example, flounder is a demersal species and during the summer age-1 pollock are found near the bottom of the water column more often than age-0 and age-2 pollock [36], [37]. Because of their vertical distribution and small body size age-1 individuals may be more vulnerable to flounder compared to other pollock life stages. This could explain the higher quantities of age-1 pollock found in flounder diets relative to other age classes (Fig. 6). In addition, the distribution and movements of juvenile pollock are influenced by water temperature [this study, 17, 18]. Kotwicki and others [18] found that with increasing temperatures, juvenile pollock migrate northward, northwestward, and shoreward, possibly to better feeding areas. Therefore, the availability of juvenile pollock to flounder in the southern and northern regions of the shelf may shift throughout the summer. We note that northward pollock migrations are also likely to outpace the summer bottom trawl survey, which proceeds from the southeast to the northwest. Therefore, it is important to be cautious when interpreting seasonal survey estimates of species occurrence, biomass, and feeding habits across space as they may be biased.

Our study indicates that shifts in the location and intensity of species overlap may be strongly influenced by flounder habitat expansion. The model predictions of increased overlap in the northwest and middle shelf regions are consistent with predictions of flounder distribution. Multiple authors have shown that density-dependent and density-independent factors affect spatial distribution of this species [11], [12]. Flounder occupancy increases northward and onto the middle shelf when their biomass is high and the cold pool is reduced [8]. The increased occupancy of flounder in recent years may be driven by increased competition for space and resources in typical high-density areas, i.e. the outer continental shelf and slope. The findings of Ciannelli and others [8] and Zador and others [12] suggest that flounder might have reached their carrying capacity in some areas of the shelf and slope. Thus, flounder may move into marginal habitats to seek out better feeding conditions, or may follow the movement of their prey. Our exploratory analysis indicated that the spatial distributions of flounder and pollock were not strongly influenced by one another. There was only a slight improvement in model fits when we included spatially explicit terms for juvenile pollock and adult flounder occurrence in the other’s model (Table S1). More work is needed to identify whether the temporal and spatial scales of the data influence this outcome. However, there can still be a strong interaction between these species even if they do not have a large effect on each other’s spatial distribution.

The expansion of a predator population into new habitat can shift species dominance, particularly if the predator imposes increased mortality on the young stages of heavily exploited populations. A shift in dominance between flounder and pollock is not unprecedented. Flounder replaced pollock as the dominant groundfish species in the Gulf of Alaska ecosystem in the early 1990s, and now controls pollock recruitment there [38]. Previous studies indicate that heavy exploitation coupled with increased predation by flounder exacerbated the decline of Gulf of Alaska pollock [39], [40]. The eastern Bering Sea pollock population size has remained fairly high and stable over the past few decades [16]. However, the flounder population in this system is on a similar trajectory to that observed in the Gulf of Alaska.

The mechanisms that regulate the flounder population in the eastern Bering Sea are essentially unknown. There is only a minor fishery for flounder in the northeast Pacific Ocean and there is little evidence of flounder in predator diets. Wilderbuer and others [41] suggest that the control of flounder production is primarily based on physical drivers. However, biological drivers might play a role as well. For example, adult pollock are a primary predator of juvenile flounder and might limit flounder population growth [13]. The complex web of trophic interactions between pollock and flounder makes them likely to experience nonlinear dynamics that are difficult to reverse, if flounder biomass and their predation impact on juvenile pollock increases. We note that by including annual adult pollock (age 3+) biomass in the flounder model the model fit was improved, but subsequent analyses based on that model essentially produced the same results and conclusions as the best fit model without adult pollock (equation 1). Our impression is that the effect of adult pollock in the flounder model is related to some common forcing of temperature and biomass on flounder distribution. However, there could be multiple mechanisms at play (e.g. predation, competition) that warrant investigation in future studies.

Pollock provide sustenance for many species of commercial and conservation value, and support the world’s second largest single-species commercial fishery. Increased predation on juvenile stages combined with other top-down [31], [35], [42]–[43] and bottom-up [35], [42], [44]–[46] forcing on the survival of pollock early life stages could have important ecological and economic consequences. More work is needed to quantify the interaction strengths of juvenile pollock and flounder, and other predators in the eastern Bering Sea under different environmental and fishing scenarios.

## Supporting Information

Figure S1 Estimation of threshold flounder biomass. Akaike information criterion (AIC) levels with different estimates of flounder biomass in full model formulation (see Model 5 in Table 1). Actual estimates of flounder biomass (in tons) are shown in addition to standardized estimates (see Methods).
(TIF)

Figure S2 Probability of species occurrence. The predicted probability of occurrence of juvenile pollock (A) and flounder (B) averaged over all years based on the best fit GAM selected for each species (see Table 1). Light and dark colors indicate locations of highest and lowest occurrence, respectively. The 50 m, 100 m and 200 m depth contours are shown. Inner shelf, <50 m; middle shelf, 50 to 100 m; outer shelf, >100 m
(TIF)

Figure S3 Temporal change in correlations between residuals of a pollock stock-recruitment relationship and biotic and abiotic indices. Correlation coefficients were calculated for 15-year moving windows centered at the x-axis values. The symbols indicate the annual mean flounder and pollock overlap (Overlap_yr−1_), ln-transformed flounder stock size (Flounder_yr−1_), and average summer trawl survey bottom (gear) temperatures (BT_yr−1,_ °C). Stippled lines: statistical significance of correlations = 0.05 (ignoring autocorrelation).
(TIF)

Table S1 Additional versions of GAMs. Full models from Table 1 that also include terms for juvenile pollock and flounder occurrence in the other’s model. We did not include these models in our list of candidate models because the inclusion of the predator and prey terms could have confounding effects on the estimates of species overlap and the recruitment analysis.
(DOCX)

Table S2 The statistical significance of all variables in the best fit pollock and flounder GAMs. See Methods for description of variables. The estimated degrees of freedom are shown for smooth terms and linear coefficients and standard errors are shown for parametric.
(DOCX)
