# Supplementary figures and images for: Climate and Demography Dictate the Strength of Predator-Prey Overlap in a Subarctic Marine Ecosystem
Source: PLoS One. 2013 Jun 18;8(6):e66025. doi: 10.1371/journal.pone.0066025 (PMC3688855; doi:10.1371/journal.pone.0066025)

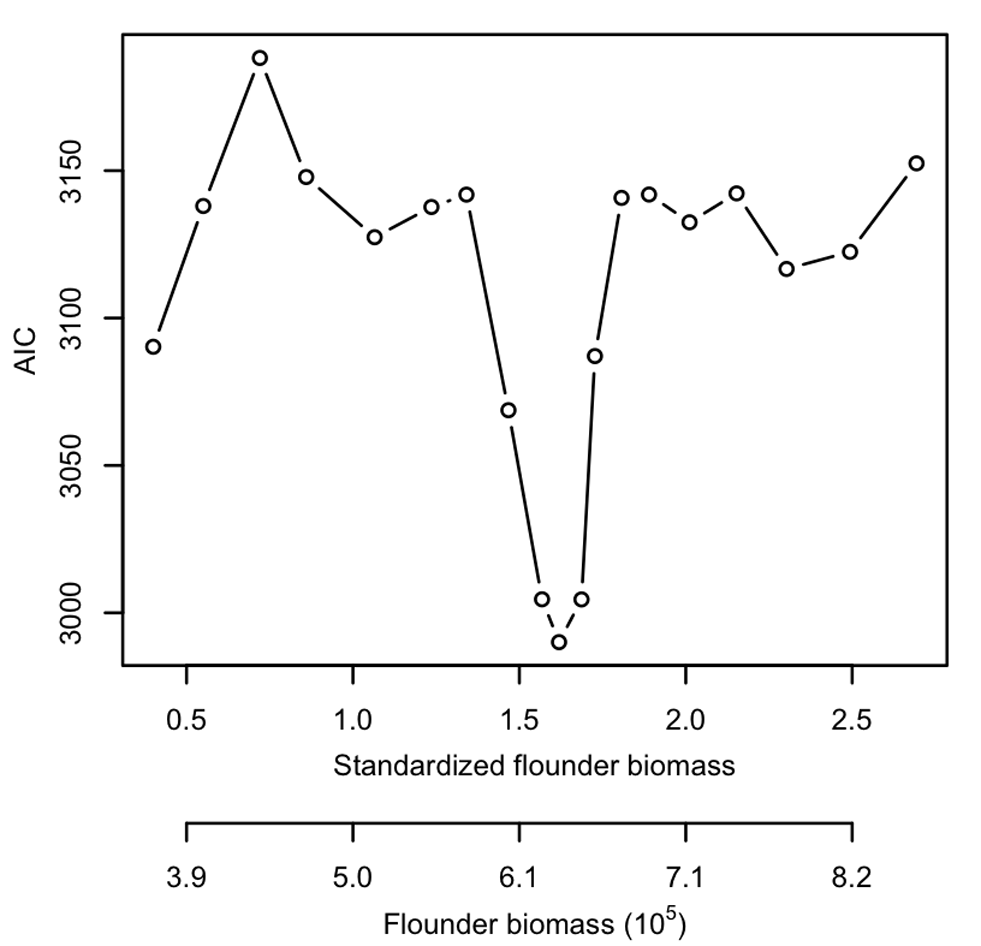

Supplement: Figure S1 — Estimation of threshold flounder biomass. Akaike information criterion (AIC) levels with different estimates of flounder biomass in full model formulation (see Model 5 in Table 1). Actual estimates of flounder biomass (in tons) are shown in addition to standardized estimates (see Methods). (TIF) [file pone.0066025.s001.tif]

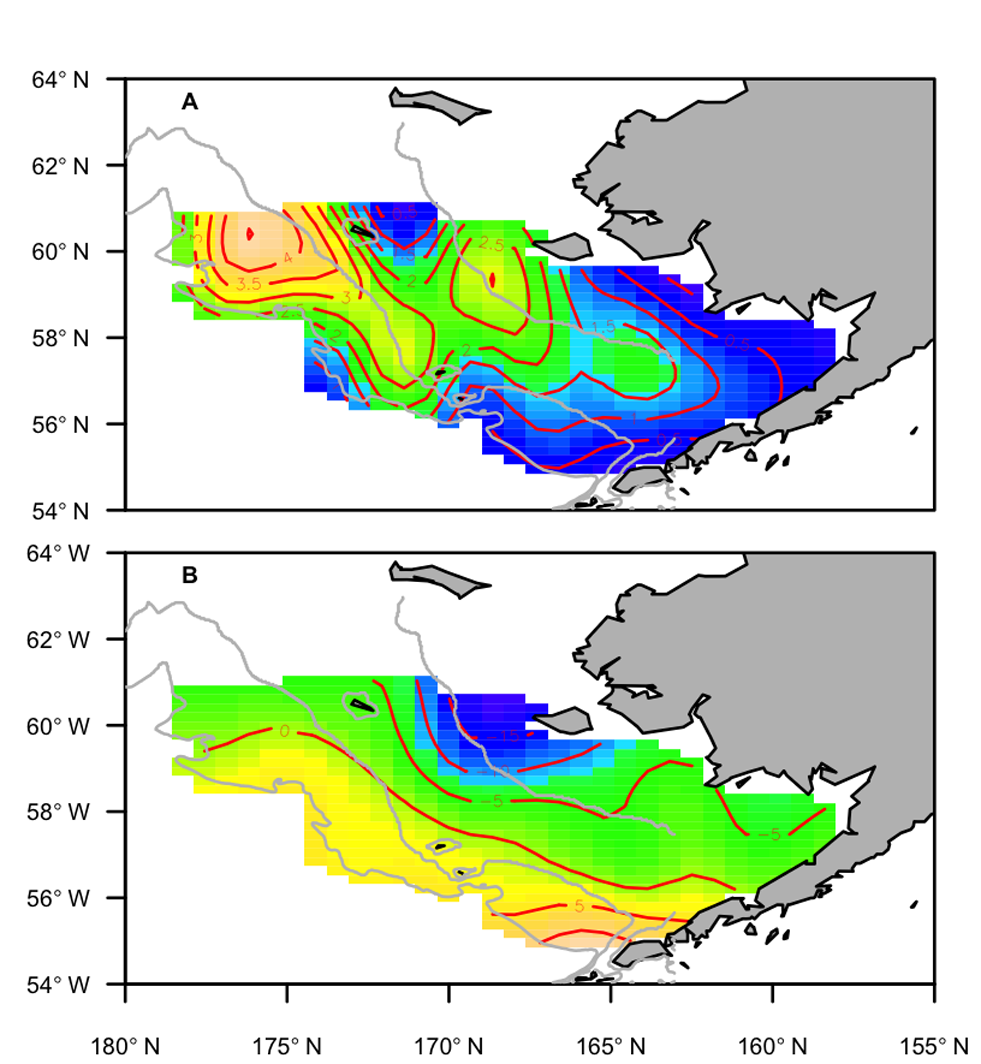

Supplement: Figure S2 — Probability of species occurrence. The predicted probability of occurrence of juvenile pollock (A) and flounder (B) averaged over all years based on the best fit GAM selected for each species (see Table 1). Light and dark colors indicate locations of highest and lowest occurrence, respectively. The 50 m, 100 m and 200 m depth contours are shown. Inner shelf, <50 m; middle shelf, 50 to 100 m; outer shelf, >100 m (TIF) [file pone.0066025.s002.tif]

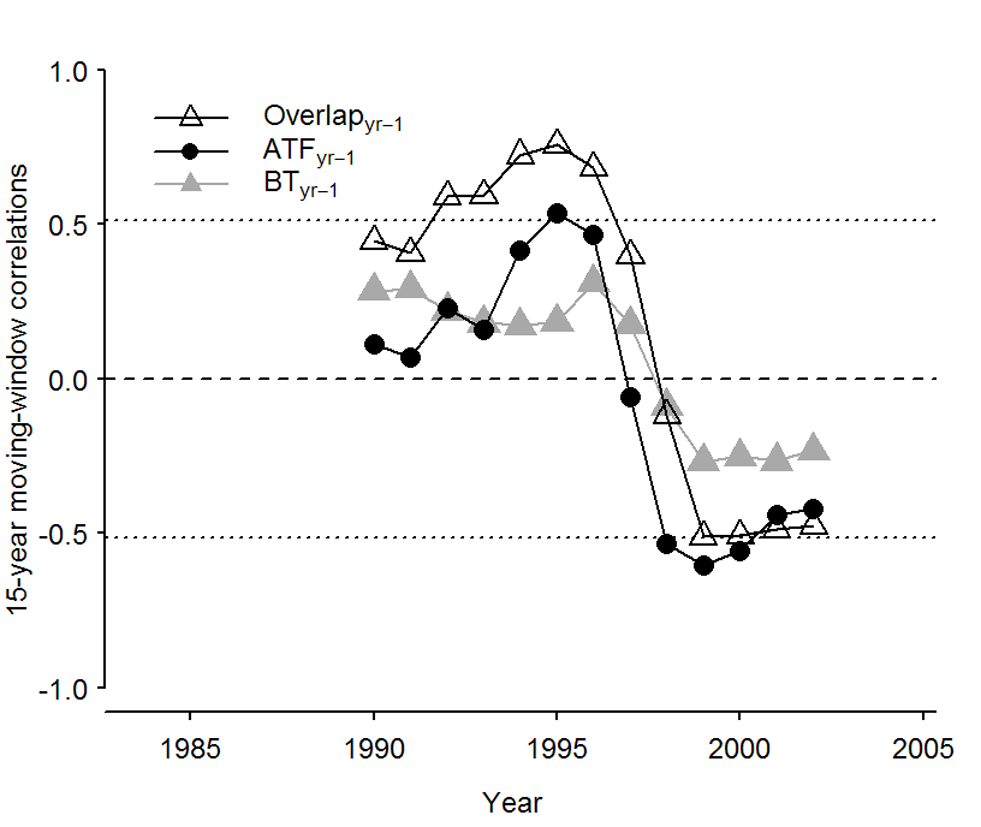

Supplement: Figure S3 — Temporal change in correlations between residuals of a pollock stock-recruitment relationship and biotic and abiotic indices. Correlation coefficients were calculated for 15-year moving windows centered at the x-axis values. The symbols indicate the annual mean flounder and pollock overlap (Overlapyr−1), ln-transformed flounder stock size (Flounderyr−1), and average summer trawl survey bottom (gear) temperatures (BTyr−1, °C). Stippled lines: statistical significance of correlations = 0.05 (ignoring autocorrelation). (TIF) [file pone.0066025.s003.tif]

| **ID** | **Models** | **Dev expl** | **UBRE** | **AIC** | **gCV** |
| --- | --- | --- | --- | --- | --- |
|  | *Juvenile pollock* |  |  |  |  |
| 1A | 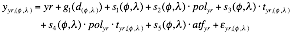 | 21.3 | -0.035 | 7323 | 0.1588 |
| 2A | 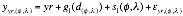 | 19.5 | -0.025 | 7394 | 0.1602 |
| 3A | 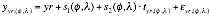 | 19.9 | -0.024 | 7402 | 0.1604 |
| 4A | 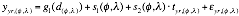 | 16.4 | -0.011 | 7669 | 0.1677 |
|  | *Arrowtooth flounder* |  |  |  |  |
| 5A | 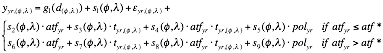 | 74.2 | -0.609 | 2969 | 00617 |
| 6A | 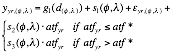 | 62.5 | -0.471 | 4013 | 0.0847 |
| 7A | 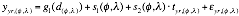 | 59.4 | -0.432 | 4310 | 0.0884 |
| 8A | 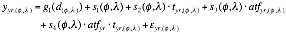 | 70.3 | -0.574 | 3226 | 0.0646 |

Supplement: Table S1 — Additional versions of GAMs. Full models from Table 1 that also include terms for juvenile pollock and flounder occurrence in the other’s model. We did not include these models in our list of candidate models because the inclusion of the predator and prey terms could have confounding effects on the estimates of species overlap and the recruitment analysis. (DOCX) [file pone.0066025.s004.docx]
