# Supplementary material for: Climate and Demography Dictate the Strength of Predator-Prey Overlap in a Subarctic Marine Ecosystem
Source: PLoS One. 2013 Jun 18;8(6):e66025. doi: 10.1371/journal.pone.0066025 (PMC3688855; doi:10.1371/journal.pone.0066025)
Supplement: Table S2 — The statistical significance of all variables in the best fit pollock and flounder GAMs. See Methods for description of variables. The estimated degrees of freedom are shown for smooth terms and linear coefficients and standard errors are shown for parametric. (DOCX) [file pone.0066025.s005.docx]

|  |  | **edf** | **Chi Sq** | **Nominal p-vaue** |
| --- | --- | --- | --- | --- |
| Arrowtooth flounder |  |  |  |  |
| 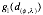 |  | 7.148 | 61.96 | <0.001 |
| 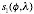 |  | 9.164 | 43.18 | <0.001 |
| 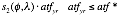 |  | 16.755 | 43.68 | <0.01 |
| 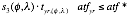 |  | 4.912 | 17.22 | <0.001 |
| 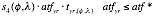 |  | 24.368 | 83.14 | <0.01 |
| 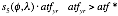 |  | 16.468 | 57.3 | <0.001 |
| 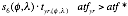 |  | 26.721 | 80.84 | <0.001 |
| 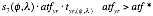 |  | 29.986 | 136.63 | <0.001 |
|  |  |  |  |  |
| Juvenile pollock |  |  |  |  |
| 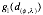 |  | 4.951 | 36.45 | <0.001 |
| 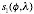 |  | 18.922 | 92.17 | <0.001 |
| 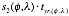 |  | 29.158 | 65.38 | <0.001 |
|  | **Estimate** | **Std. Error** | **z value** | **Pr (>\|z\|)** |
| Arrowtooth flounder |  |  |  |  |
| *(Intercept)* | -6.052 | 0.848 | -7.134 | <0.001 |
| Juvenile pollock |  |  |  |  |
| *(Intercept)* | 1.320 | 0.246 | 5.363 | <0.001 |
| *yr (1983)* | -0.637 | 0.211 | -3.020 | <0.01 |
| *yr (1984)* | -0.611 | 0.209 | -2.920 | <0.01 |
| *yr (1985)* | 0.236 | 0.228 | 1.038 |  |
| *yr (1986)* | 0.714 | 0.230 | 3.111 | <0.01 |
| *yr (1987)* | -1.529 | 0.210 | -7.280 | <0.001 |
| *yr (1988)* | -0.696 | 0.207 | -3.358 | <0.001 |
| *yr (1989)* | -1.013 | 0.206 | -4.912 | <0.001 |
| *yr (1990)* | 0.194 | 0.234 | 0.829 |  |
| *yr (1991)* | 0.356 | 0.232 | 1.537 |  |
| *yr (1992)* | -0.256 | 0.220 | -1.166 |  |
| *yr (1993)* | -0.472 | 0.214 | -2.199 | <0.05 |
| *yr (1994)* | -0.126 | 0.221 | -0.573 |  |
| *yr (1995)* | 0.114 | 0.220 | 0.517 |  |
| *yr (1996)* | 0.753 | 0.242 | 3.113 | <0.01 |
| *yr (1997)* | -0.002 | 0.217 | -0.008 |  |
| *yr (1998)* | 0.318 | 0.229 | 1.392 |  |
| *yr (1999)* | 0.443 | 0.229 | 1.935 | 0.100 |
| *yr (2000)* | 0.110 | 0.217 | 0.509 |  |
| *yr (2001)* | 0.472 | 0.226 | 2.091 | <0.05 |
| *yr (2002)* | 0.043 | 0.219 | 0.198 |  |
| *yr (2003)* | -0.972 | 0.214 | -4.536 | <0.001 |
| *yr (2004)* | -0.744 | 0.211 | -3.526 | <0.001 |
| *yr (2005)* | -0.582 | 0.219 | -2.654 | <0.01 |
| *yr (2006)* | 0.230 | 0.218 | 1.054 |  |
| *yr (2007)* | 0.572 | 0.227 | 2.513 | <0.05 |
| *yr (2008)* | -0.251 | 0.212 | -1.184 |  |
